# Cutaneous Metastatic Cancer: Carcinoma Hemorrhagiectoides Presenting as the Shield Sign

**DOI:** 10.7759/cureus.12627

**Published:** 2021-01-11

**Authors:** Philip R Cohen, Razelle Kurzrock

**Affiliations:** 1 Dermatology, San Diego Family Dermatology, National City, USA; 2 Center for Personalized Cancer Therapy, University of California San Diego Moores Cancer Center, La Jolla, USA

**Keywords:** breast, cancer, carcinoma, cutaneous, erysipelatoides, hemorrhagiectoides, metastatic, shield, sign, telangiectoides

## Abstract

Cutaneous metastases can be either the initial presentation of an unsuspected internal neoplasm in a cancer-free individual, or the harbinger of recurrent malignancy in an oncology patient who had achieved remission of his cancer, or a sign of progressive disease in a cancer patient who is currently receiving antineoplastic treatment. The cutaneous presentation of skin metastases is pleomorphic and can mimic not only benign conditions and tumors of the skin but also infections and inflammation of the skin. Carcinoma erysipelatoides, carcinoma hemorrhagiectoides, and carcinoma telangiectoides are the three subtypes of inflammatory cutaneous metastatic cancer. The former masquerades as a cutaneous streptococcal infection whereas the latter mimics idiopathic telangiectasias. In contrast, the morphology of carcinoma hemorrhagiectoides is distinctive: it appears similar in shape to a medieval knight’s shield and its presence is referred to as a positive shield sign. To the best of our knowledge, carcinoma hemorrhagiectoides has been reported in four oncology patients whose skin metastases presented with the shield sign: two men with salivary duct carcinoma and two women with breast cancer. In conclusion, the shield sign may not only be a pathognomonic clinical feature of carcinoma hemorrhagiectoides but also reflect a common genomic aberration of these metastatic tumors.

## Introduction and background

Cutaneous metastases do not occur frequently. Typically, when they do appear, it is in the setting of an oncology patient whose visceral malignancy is progressing. However, albeit less often, they may be either the initial presentation of malignancy in a previously cancer-free individual or the heralding sign of cancer recurrence in a patient whose malignancy had been in remission [1-19].

The morphology of cutaneous metastases is variable. In addition to cutaneous conditions and benign neoplasms, skin metastases can mimic infection diseases [3-7]. Cutaneous metastases can also present with inflammatory patterns of skin involvement [8,9].

Carcinoma hemorrhagiectoides is a subtype of inflammatory cutaneous metastases [9-11]; it has a distinctive morphologic presentation, similar to a medieval knight’s shield, on the chest [9]. Prior to the description of carcinoma hemorrhagiectoides as a specific variant of inflammatory cutaneous metastases, the skin manifestations of patients whose cutaneous metastases had this particular clinical appearance may have been classified as either carcinoma erysipelatoides or carcinoma telangiectoides. The appearance of cutaneous metastases is summarized and the characteristics of patients whose metastatic skin lesions presented as carcinoma hemorrhagiectoides with the shield sign are reviewed.

## Review

Pleomorphic presentation of cutaneous metastases

The morphologic presentation of cutaneous cancer metastases can mimic benign skin tumors, dermatologic conditions, and vascular lesions. The nodular lesions of metastatic carcinoma can mimic dermatofibromas. Metastatic carcinoma can also appear similar to dermatitis (in patients with inframammary crease skin metastases and Paget disease), hair loss (in patients with alopecia neoplastica), periobital edema (in patients with painless eyelid swelling), and scleroderma (in patients with carcinoma en cuirasse) [3,4].

Specific tumor-associated cutaneous metastases morphologies

Cutaneous metastases from specific cancers can be variable in their presentations. Breast cancer metastases to the skin commonly present as nodules. However, they can appear as bullae, inflammatory carcinoma metastases (erysipelatoides, hemorrhagiectoides, and telangiectoides), or plaques [3-5,8,12].

Cutaneous breast metastases can also mimic other conditions (such as alopecia, dermatitis, granuloma annulare, herpes zoster, hidradenitis suppurativa, and scleroderma) or tumors (such as keratoacanthoma and melanoma). They are most frequently found on the skin of the ipsilateral affected breast. However, they can be located on the opposite breast skin, inframammary fold, periocular area, scalp, subungual region, and umbilicus [3-5,8,12].

In contrast to breast cancer cutaneous metastases, other malignant neoplasms, such as metastatic neuroblastoma, have fewer clinical presentations of cancer-associated skin metastases that have been observed. Neuroblastoma cutaneous metastases typically present as blue-gray nodules that blanch after stroking. However, they also can appear as periorbital ecchymoses and heterochromic iridis [13].

Salivary duct carcinoma is a rare malignancy derived from the parotid glands; to our best knowledge, only six salivary duct carcinoma patients with cutaneous metastases have been described: five men and one woman [9,14-19]. Two of the men, whose clinical features of their cutaneous metastases have been described in this review, had carcinoma hemorrhagiectoides. Their skin metastases presented as purpuric, hemorrhagic, and erythematous dermal plaques with either bullae and pseudovesicles or black, keratotic angiokeratoma-like papules [9,15].

Two of the other men’s salivary duct carcinoma skin metastases presented as subcutaneous nodules that were either skin-colored on the scapula and suprapatellar areas or zosteriform and linearly distributed on the chest and neck [16-18]. The fifth man’s salivary duct carcinoma cutaneous metastasis presented as a superficial ulcer on his forehead mimicking a basal cell carcinoma and was treated by curettage; the non-healing wound was excised and the diagnosis of salivary duct carcinoma skin metastasis was established [14]. The woman’s salivary duct carcinoma metastases to the skin initially appeared as red macules on her cheek and preauricular area; purpuric papules and pseudovesicles on the preauricular area and edema of her eyes developed subsequently [19].

Cutaneous metastases masquerading as infection or other conditions

Skin metastases can masquerade as bacterial infections such as cellulitis (in patients with carcinoma erysipelatoides) or acute paronychia. They can also occur in a zosteriform distribution similar to varicella zoster virus infection. In addition, the metastatic cancer can appear as granulation tissue (pyogenic granuloma-like), or palpable purpura (mimicking vasculitis), or similar lymphangioma circumscriptum (in patients with carcinoma telangiectoides) [5-7].

Inflammatory cutaneous metastases

There are three variants of inflammatory cutaneous metastatic cancer: carcinoma erysipelatoides, carcinoma hemorrhagiectoides, and carcinoma telangiectoides (Table 1) [8,9].

**Table 1:** Characteristics of inflammatory cutaneous metastatic carcinoma.

Feature	Carcinoma erysipelatoides	Carcinoma hemorrhagiectoides	Carcinoma telangiectoides
Cutaneous morphology	Patch or plaque: erythematous and sharply demarcated	Indurated plaque: purpuric and violaceous	Patch: erythematous with prominent telangiectasias
Presence of tumor in the dermis	0% to 3-5%; absent to minimal	6% to >25%; mild to extensive	0% to 3-5%; absent to minimal
Vessel infiltrated by the tumor	Lymphatics	Blood vessels or lymphatics or both	Blood vessels

Carcinoma erysipelatoides

Carcinoma erysipelatoides presents as a sharply demarcated erythematous patch or plaque. It mimics a streptococcal infection of the skin. The tumor cells infiltrate the lymphatic vessels and are minimally present (or absent) in the dermis [8,9].

Carcinoma telangiectoides

Carcinoma telangiectoides presents as an erythematous patch with prominent telangiectasias. It can mimic idiopathic telangiectasias. However, the tumor cells usually infiltrate the blood vessels and are minimally present (or absent) in the dermis [8,9].

Carcinoma hemorrhagiectoides

**Figure 1:**
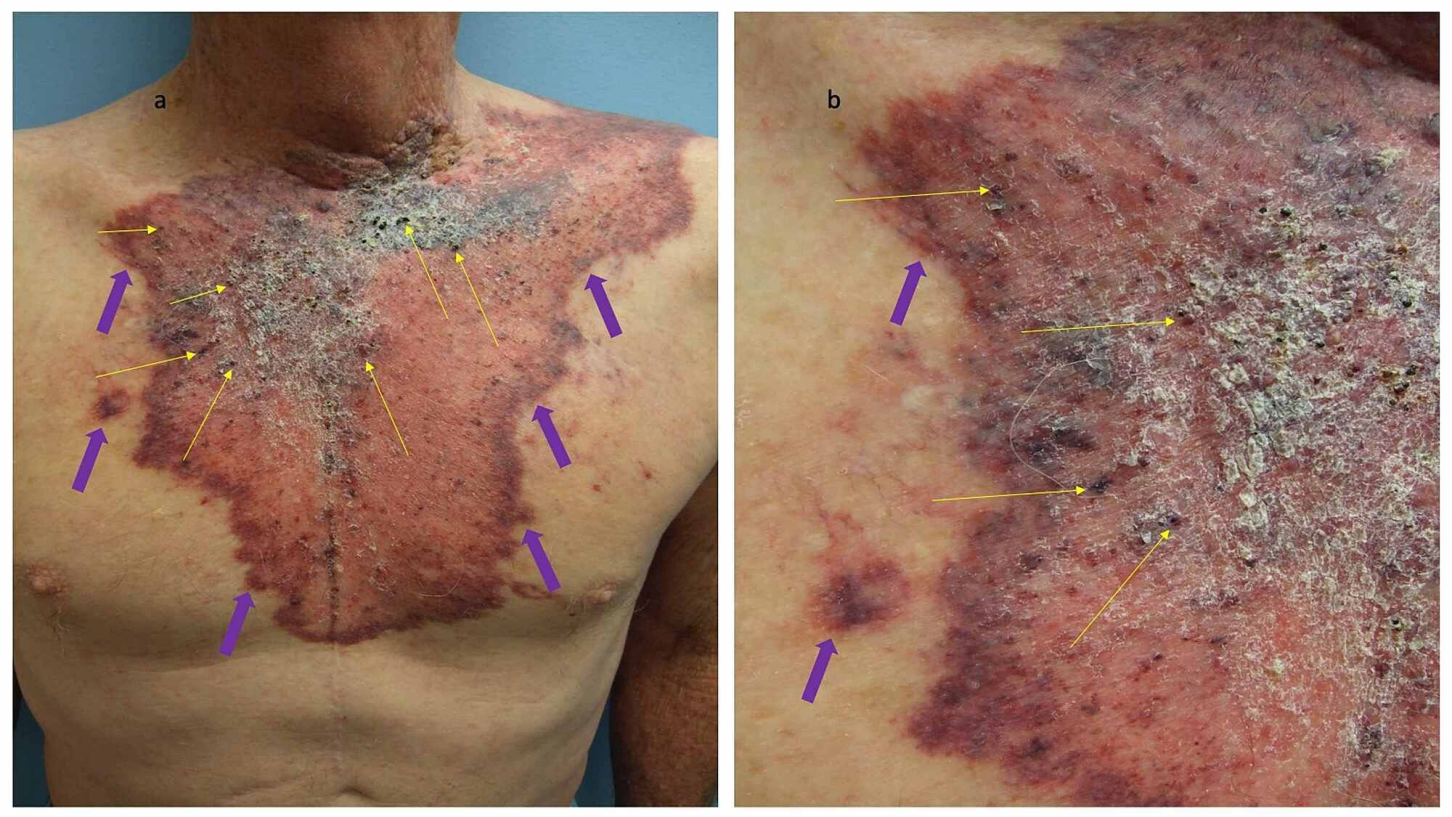
Cutaneous metastatic salivary duct carcinoma-associated carcinoma hemorrhagiectoides presenting as the shield sign. A 73-year-old man presented 17 months earlier with metastatic salivary duct tumor; following a left radical parotidectomy and modified radical neck dissection, he received chemoradiation (60 Gray in 30 fractions and weekly cisplatin). His tumor recurred with cutaneous metastases. Distant (a) and closer (b) views of his cutaneous metastatic salivary duct carcinoma appeared as a hemorrhagic and erythematous confluent large violaceous and purpuric plaque (purple arrows) with angiokeratoma-like black keratotic papules (yellow arrows) that extended from his neck to his abdomen and across his chest, resembling a medieval knight’s shield. Biopsy of the cutaneous metastatic lesion showed tumor cells within lymphatic vessels and extensively throughout the dermis. The details of this patient’s skin lesion have previously been described (Cohen et al., 2012).

Carcinoma hemorrhagiectoides was initially described in 2012 in two men with metastatic salivary duct carcinoma (Table 2) [9-11]. In addition, the distribution of the cutaneous metastases in both of these patients was distinctive: it resembled a medieval knight’s shield (Figure 1) [9]. In contrast to carcinoma erysipelatoides and carcinoma telangiectoides, carcinoma hemorrhagiectoides presents as violaceous, purpuric, indurated plaques; the tumor cells infiltrate the blood vessels or the lymphatic vessels or both and they are mildly to extensively present in the dermis [9-11].

**Table 2:** Characteristics of patients with carcinoma hemorrhagiectoides. A, age (in years); Abd, abdomen; Adeno, adenocarcinoma; Ak, angiokeratoma; C, case; CM, cutaneous metastasis; Ery, erythematous; Hem, hemorrhagic; ID, intradermal; IDC, invasive ductal carcinoma; IL, intralymphatic; IV, intravascular (blood vessel); M, man; Morph, morphology; NS, not stated; P Sh, posterior shoulder; Path, pathology; Pres, presentation; Prog Dz, progressive disease; Psvesic; pseudovesicle; Purp, purpuric; Recur, recurrence; Ref, reference; S, sex; Scap, scapula; SC, subcutis (fat); SDC, salivary duct carcinoma; Sh, shoulder; Viol, violaceous; W, woman; -, absent; + present ^a^This is the patient’s age when the cutaneous metastasis appeared. ^b^These are the other sites, in addition to the chest and breasts, where the cutaneous metastases were also present. ^c^These are the locations where the metastatic carcinoma was present or absent in the skin. ^d^His cancer presented 11 months earlier when he was 69 years old. He developed additional lesions within two months. Two months after receiving five cycles of docetaxel and chemoradiation, he presented with progressive disease including the cutaneous metastases. ^e^At 71 years of age, he presented with metastatic cancer; he had surgery followed by chemoradiation. His cutaneous metastases were the harbinger of his recurrent tumor 11 months later. ^f^At 79 years of age, she was diagnosed with stage IV breast cancer that was metastatic to her chest wall. She had a bilateral mastectomy. A skin eruption began a few months postoperatively. Her cutaneous metastases continue to progress during the next two years until she was evaluated. ^g^At 90 years of age, she presented to the emergency room for evaluation of her chest wall lesions of unknown evolution. Hence, her cutaneous metastases were the initial presentation of her previously undiagnosed breast cancer. Systemic evaluation demonstrated extensive nodal disease. She refused treatment and died at home a month and a half later.

C	A^a^ S	Cancer origin	Cancer type	Onset of CM	Shield sign	Other sites of CM^b^	Color of CM	Morph of CM	Path of CM^c^	Ref
1	70 M	Left parotid	SDC	Prog Dz^d^	+	Abd, Face, Neck	Hem, Purp, Viol	Bullae, Plaque, Psvesic	ID: +, IL: +, IV: +, SC: NS	[9] C2
2	73 M	Left parotid	SDC	Recur^e^	+	Abd, Neck, P Sh	Ery, Hem, Purp, Viol	Ak-like, Papules, Plaque	ID: +, IL: +, IV: NS, SC: NS	[9] C1
3	81 W	Breasts	Adeno	Prog Dz^f^	+	Abd, Arm, Back, Flank	Ery, Purp, Viol	Nodules, Papule, Patch, Psvesic	ID: +, IL: +, IV: NS, SC: NS	[11]
4	90 W	Left breast	IDC	Initial Pres^g^	+	Arm, Back, Scap, Sh	Ery, Purp	Plaque	ID: +, IL: +, IV: NS, SC: +	[10]

Subsequently, in 2017, a 90-year-old woman with invasive ductal breast carcinoma was reported whose cutaneous metastases presented as carcinoma hemorrhagiectoides. Her metastatic breast cancer skin lesions appeared as a well-circumscribed, indurated, and infiltrated plaque involving the left breast, ipsilateral chest wall, shoulder, arm, and scapular region with spread beyond the midline toward the right breast; the plaque was not only erythematous but also had areas that were more violaceous. Microscopic examination showed emboli of breast carcinoma tumor cells in the lumina of dermal lymphatic vessels; in addition, there was an abundance of metastatic tumor cells in the dermis with extension into the subcutaneous fat [10].

Recently, in 2020, an 81-year-old woman with breast adenocarcinoma developed cutaneous metastases on the chest, abdomen, flank, and back that presented as carcinoma hemorrhagiectoides. Her purpuric grouped skin lesions appeared on a background of violaceous erythema; they were firm papules, nodules, and pseudovesicles. Microscopic examination of the cutaneous metastasis not only showed intralymphatic invasion by the tumor cells but also abundant metastatic breast carcinoma cells throughout the dermis. The combination of tumor cells in both the lymphatic vessel and within the dermis resulted in the hemorrhagic infiltrated plaque-like appearance of the violaceous patches with overlying purpuric papules, nodules, and pseudovesicles observed in the images of her cutaneous metastases [11].

In summary, carcinoma hemorrhagiectoides has been reported in four cancer patients whose skin metastases presented with the shield sign. Two of the patients were men with salivary duct carcinoma that originated in their parotid gland. The other two patients were women with either adenocarcinoma of the breast or invasive ductal breast carcinoma (Table 2) [9-11].

The skin lesions represented either progressive neoplastic disease (two patients), cancer recurrence (one man), or initial presentation of malignancy (one woman). The erythematous, purpuric, and violaceous color of the metastatic infiltrated plaque or macule produced a hemorrhagic appearance; angiokeratoma-like lesions, bullae, papules, and pseudovesicles were also present. In addition to the chest, all patients had concurrent skin metastases at other sites [9-11].

Microscopic examination demonstrated tumor cells throughout the dermis. In addition, tumor cells were also within lymphatic vessels. Some of the patients also had tumor cells in blood vessels or the subcutaneous fat [9-11].

In general, the appearance of cutaneous metastases is not a favorable development for a cancer patient. However, both of the men with salivary duct carcinoma demonstrated a dramatic improvement of their neoplastic disease after receiving treatment with temsirolimus and bevacizumab; yet, both men died shortly after initiating treatment: the 70-year-old man from a myocardial infarction and the 73-year-old man from progressive disease after stopping therapy [9,15]. The 81-year-old woman elected to continue receiving homeopathic anticancer shakes and was lost to follow-up, whereas the 90-year-old woman’s neoplastic disease was not treated and she died within one and a half months after diagnosis [10,11].

## Conclusions

Carcinoma hemorrhagiectoides is a distinctive morphologic presentation of cutaneous metastatic carcinoma. The clinical presentation of papules with an underlying indurated violaceous plaque correlates with the pathology findings of tumor infiltration not only in vessels-blood, lymphatic, or both-but also extensively throughout the dermis. To date, carcinoma hemorrhagiectoides has only been observed in breast cancer patients with metastatic adenocarcinoma or invasive ductal carcinoma, as well as in oncology patients with metastatic salivary duct carcinoma. Similar to the men with salivary duct carcinoma, the distribution of the cutaneous metastases in the women with breast cancer also resembled a medieval knight’s shield that was extending across their chests. We suggest that the shield sign may be a pathognomonic clinical feature of carcinoma hemorrhagiectoides and speculate that it may reflect a common genomic aberration of these tumors.
